# Co-occurrence of Beckwith-Wiedemann syndrome and pseudohypoparathyroidism type 1B: coincidence or common molecular mechanism?

**DOI:** 10.3389/fcell.2023.1237629

**Published:** 2023-08-10

**Authors:** Laura Pignata, Francesco Cecere, Fabio Acquaviva, Emilia D’Angelo, Daniela Cioffi, Valeria Pellino, Orazio Palumbo, Pietro Palumbo, Massimo Carella, Angela Sparago, Daniele De Brasi, Flavia Cerrato, Andrea Riccio

**Affiliations:** ^1^ Department of Environmental Biological and Pharmaceutical Sciences and Technologies (DiSTABiF), Università degli Studi della Campania “Luigi Vanvitelli”, Caserta, Italy; ^2^ UOSD Genetica Medica, Dipartimento di Pediatria Generale e d’Urgenza, AORN Santobono-Pausilipon, Naples, Italy; ^3^ UOSD Auxologia e Endocrinologia Pediatrica, Dipartimento di Pediatria Specialistica, AORN Santobono-Pausilipon, Naples, Italy; ^4^ Division of Medical Genetics, Fondazione IRCCS “Casa Sollievo della Sofferenza”, San Giovanni Rotondo, Italy; ^5^ Istituto di Genetica e Biofisica “Adriano Buzzati Traverso” Consiglio Nazionale delle Ricerche, Naples, Italy

**Keywords:** Beckwith-Wiedemann syndrome, pseudohypoparathyroidism type 1B, multilocus imprinting disturbance, uniparental disomy, imprinting disorders, genomic imprinting, *KCNQ1OT1*:TSS-DMR, *GNAS*

## Abstract

Imprinting disorders are congenital diseases caused by dysregulation of genomic imprinting, affecting growth, neurocognitive development, metabolism and cancer predisposition. Overlapping clinical features are often observed among this group of diseases. In rare cases, two fully expressed imprinting disorders may coexist in the same patient. A dozen cases of this type have been reported so far. Most of them are represented by individuals affected by Beckwith–Wiedemann spectrum (BWSp) and Transient Neonatal Diabetes Mellitus (TNDM) or BWSp and Pseudo-hypoparathyroidism type 1B (PHP1B). All these patients displayed Multilocus imprinting disturbances (MLID). Here, we report the first case of co-occurrence of BWS and PHP1B in the same individual in absence of MLID. Genome-wide methylation and SNP-array analyses demonstrated loss of methylation of the *KCNQ1OT1*:TSS-DMR on chromosome 11p15.5 as molecular cause of BWSp, and upd(20)pat as cause of PHP1B. The absence of MLID and the heterodisomy of chromosome 20 suggests that BWSp and PHP1B arose through distinct and independent mechanism in our patient. However, we cannot exclude that the rare combination of the epigenetic defect on chromosome 11 and the UPD on chromosome 20 may originate from a common so far undetermined predisposing molecular lesion. A better comprehension of the molecular mechanisms underlying the co-occurrence of two imprinting disorders will improve genetic counselling and estimate of familial recurrence risk of these rare cases. Furthermore, our study also supports the importance of multilocus molecular testing for revealing MLID as well as complex cases of imprinting disorders.

## 1 Introduction

Imprinted genes are autosomal genes preferentially expressed from one of the two parental chromosomes. This monoallelic and parent-of-origin expression is due to the presence of differential DNA methylation between the two parental alleles of *cis*-acting elements known as imprinted differentially methylated regions (iDMRs) (Monk et al., 2019). Dysregulation of imprinted genes results in a group of congenital diseases (Imprinting Disorders, ImpDis) characterized by defective pre- and post-natal growth, neurocognitive development, metabolism, and increased cancer predisposition (Carli et al., 2020).

The Beckwith–Wiedemann spectrum (BWSp, OMIM 130650) and Pseudo-hypoparathyroidism type 1B (PHP1B, OMIM 603233) are ImpDis affecting two clusters of imprinted genes located on chromosome 11p15.5 and chromosome 20q13, respectively (Monk et al., 2019).

The clinical diagnosis of BWSp is based on the manifestation of cardinal features (e.g., macroglossia, exomphalos, lateralized overgrowth) and suggestive features (e.g., neonatal macrosomia, facial naevus flammeus, polyhydramnios, ear creases or pits, abdominal wall defects) (Brioude et al., 2018). The molecular diagnosis relies on the detection of one of the following defects affecting the 11p15.5 imprinted locus: loss of methylation (LoM) of the *KCNQ1OT1*:TSS-DMR or Imprinting Centre 2 (IC2), found in 50% of cases, mosaic paternal UPD of 11p15 (20%), gain of methylation (GoM) of the *H19/IGF2*:IG-DMR or Imprinting Centre 1 (IC1) (5%–10%), loss of function mutations of the growth inhibitor *CDKN1C* (5%), and chromosomal abnormalities in 11p15.5 (1%–5%) (Brioude et al., 2018). In about one-third of the BWSp patients with IC2 LoM methylation defects also affect iDMRs on other chromosomes, a molecular condition known as Multilocus imprinting disturbances (MLID) (Bliek et al., 2009; Brioude et al., 2018).

Diagnostic criteria for MLID have been recently proposed by Ochoa et al. (2022), according to which MLID is diagnosed when methylation abnormalities are detected at an ImpDis-associated iDMR or 2 non-ImpDis-associated iDMR, in addition to the primary ImpDis-associated epimutation. Methylation changes at multiple iDMRs due UPD are not considered MLID because of their genetic origin.”

PHP1B is characterized by end-organ resistance to several endocrine hormones, including parathyroid hormone (PTH) leading to hypocalcaemia and hyperphosphatemia, and thyroid stimulating hormone (TSH) leading to clinical or subclinical hypoparathyroidism. Occasionally, features of Albright hereditary osteodystrophy (AHO) are observed (Garin et al., 2015). At the molecular level, PHP1B is associated with methylation changes of one or more DMRs of the *GNAS* cluster, including at least the *GNAS* A/B:TSS-DMR. These methylation defects result in absence of expression of the α-subunit of the stimulatory G protein (Gs-alpha) involved in hormonal signalling pathway in renal proximal tubules. In healthy individuals, the *GNAS*-A/B:TSS-DMR, *GNAS-XL:Ex1*-DMR and *GNAS-AS1*:TSS-DMR are methylated on the maternal allele, and the *NESP:TSS*-DMR is methylated on the paternal allele. Methylation changes at one or more *GNAS* DMRs can be caused by inherited deletions, usually associated with autosomal dominant pattern of inheritance through maternal lineage or may occur sporadically without evident underlying genetic abnormality (Mantovani et al., 2018). Around 8%–10% of these sporadic cases are caused by paternal UPD of chromosome 20 [upd(20)pat] and show a paternal-specific methylation pattern on both alleles of all four *GNAS* DMRs (Mantovani et al., 2018). The upd(20)pat may affect either the long arm [upd(20q)pat] or the entire chromosome 20 (Colson et al., 2019). So far, 8 cases have been reported with upd(20)pat extended to the entire chromosome, 6 of which showing isodisomy and 2 heterodisomy (Fernández-Rebollo et al., 2010; Bastepe et al., 2011; Colson et al., 2019; Choufani et al., 2021). In another subset of PHP1B cases, a MLID profile has been identified (Maupetit-Méhouas et al., 2013). However, the exact prevalence of MLID in PHP1B is uncertain because very few cohorts of patients have been screened for MLID so far. Indeed, an incidence of MLID ranging from 0% to 38% of cases can be found in the few studies reported (Izzi et al., 2010).

A clinically relevant characteristic of imprinted disorders is the heterogeneity of the phenotype that in some cases includes atypical features. This may be caused by the extension of the molecular defect to loci other than the one typically associated with the disease. Examples are represented by cases with MLID or UPD, in particular when the UPD is extended to the whole chromosome or the whole genome in mosaicism (Eggermann and Prawitt, 2022; Grosvenor et al., 2022). In rare cases, the full clinical manifestation of two ImpDis in the same patient has been reported. The diseases most frequently co-existing with BWSp are Transient Neonatal Diabetes (TNDM) (Mackay et al., 2006a; Mackay et al., 2006b; Boonen et al., 2008) and PHP1B (Bakker et al., 2015; Sano et al., 2016; Choufani et al., 2021). All these cases, including three cases of co-occurrence of BWSp and PHP1B, displayed MLID.

Here, we describe a further case of co-occurrence of BWS and PHP1B, in which the molecular mechanisms underlying the two ImpDis appear to be independent and different from the previously reported cases.

## 2 Materials and methods

### 2.1 DNA extraction

Genomic DNA of the proband and his parents was extracted from peripheral blood leukocytes (PBL) by the salting-out procedure, and a NanoDrop spectrophotometer (NanoDrop™ 2000c Spectrophotometer, Thermo Fisher Scientific) was used to determine its concentration.

### 2.2 Methylation analysis

Methylation-Specific Multiple Ligation-Dependent Probe Amplification (MS-MLPA) was performed on 50 ng of PBL DNA by using the SALSA MS-MLPA Probemix ME030-C3 (MRC-Holland, Amsterdam, Netherlands) to analyze DNA methylation and CNVs of the 11p15-BWS/SRS region, and the SALSA MS-MLPA Probemix ME034-B1 to extend the analysis to multiple imprinted loci. The amplified products were separated by capillary electrophoresis, employing an ABI 3500 Genetic Analyzer (Applied Biosystems, Foster City, CA, United States). Data were analysed using the Coffalyser software (MRC-Holland, Amsterdam, Netherlands).

Genome-wide methylation analysis was performed on bisulphite converted PBL DNA of the proband, using the Illumina Infinium MethylationEPIC BeadChip850 (array 850k). Array data were analysed using R version 4.1.0. “idat” files were imported and the beta-values were extracted using the “champ.load” module of the “ChAMP” R package v.2.22.0. Then, BMIQ normalization was applied to normalize Type 1 and Type 2 probes employing “champ.norm” function. Methylation levels of the proband were compared with 4 unaffected controls, three males aged 18, 8, and 2 years and one female aged 15 years. The effects related to age and gender were corrected using the “champ.runCombat” function specifying the conditions as variable name. Similar methylation values were obtained before and after the correction indicating that the probes targeting the iDMRs do not show age- or gender-dependent variability. Values exceeding ±3 standard deviation and differing at least 10% from average of controls were considered as abnormal methylation changes.

Methylation array datasets presented in this study can be found in GEO repository under accession code GSE237676.

### 2.3 SNP-array

Single Nucleotide Polymorphism-Array (SNP-array) analysis was carried out on PBL DNA of the proband and his parents using CytoScan™ HD Array (Thermo Fisher Scientific, Waltham, MA, United States) and in accordance with the manufacturer’s instructions. Data were analysed using the Chromosome Analysis Suite software (ChAS, Thermo Fisher Scientific, Waltham, MA, United States) version 4.0.

## 3 Results

### 3.1 Clinical report

The proband is a 6-year and 6-month-old boy, enrolled at the Department of Pediatrics of the Santobono-Pausilipon Children’s Hospital in Naples (Italy) during routine assessment for macroglossia and umbilical hernia. He is the fourth child of healthy unrelated Italian parents with unremarkable family history.

The proband, conceived naturally, was born late preterm by caesarean section at 34th week of a pregnancy complicated by placental abruption. Birth parameters were within the normal range between 25°–50° centile, Apgar score was 6 ^at 1’^/8 ^at 5’^. He showed episodic/transient hypoglycemia, hypocalcaemia and hyaline membrane disease type 1 during the perinatal period. From 2 months of age, he underwent endocrinologic surveillance for subclinical hypothyroidism, which was treated with L-thyroxine.

BWS was suspected—and then molecularly confirmed—at 3 months of age due to the clinical history and to the evidence of macroglossia and umbilical hernia. At 3 years, a mild global developmental delay was observed. The patient underwent a program of speech and psychomotor therapy that allowed to completely resolve this problem. Physical assessments over the years report normal-to-high centile parameters for weight and height (both around 90–97°ct), head circumference within the median values (M), mild chest asymmetry (left side > right side), and mild heterometric lower limbs in length (0.7 cm left > right) and thigh circumference (∼2 cm left > right). According to Brioude et al. (2018), the BWS clinical score of the patient was estimated to be 7.

Up to 5 years of age, endocrinological parameters were within normal ranges. Afterwards, mild hypocalcemia and hyperphosphatemia with elevated PTH levels suggestive of PTH resistance were detected. A second evaluation confirmed these data and suggested further investigation including molecular testing for Pseudo-hypoparathyroidism type 1B (PHP1B), which confirmed the clinical suspicion. Supplemental calcium and activated forms of vitamin D treatment was ensured, with normalization of Ca/P metabolism (see Supplementary Table S1).

### 3.2 Molecular analysis

**FIGURE 1:**
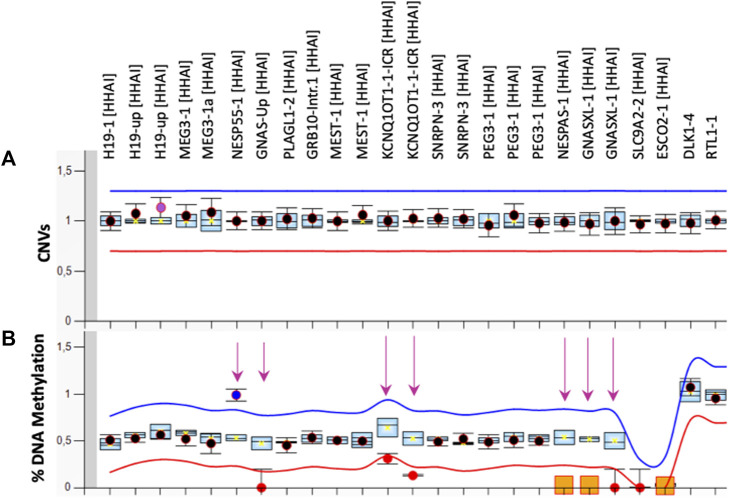
MS-MLPA results. Copy Number [CNVs, **(A)**] and DNA methylation **(B)** of 10 imprinted loci were analysed in PBL of the proband by the ME034-B1 kit. The mean values of three control subjects were used for the assessment of relative copy number and methylation percentage. The arrows indicate the methylation defects identified.

**FIGURE 2:**
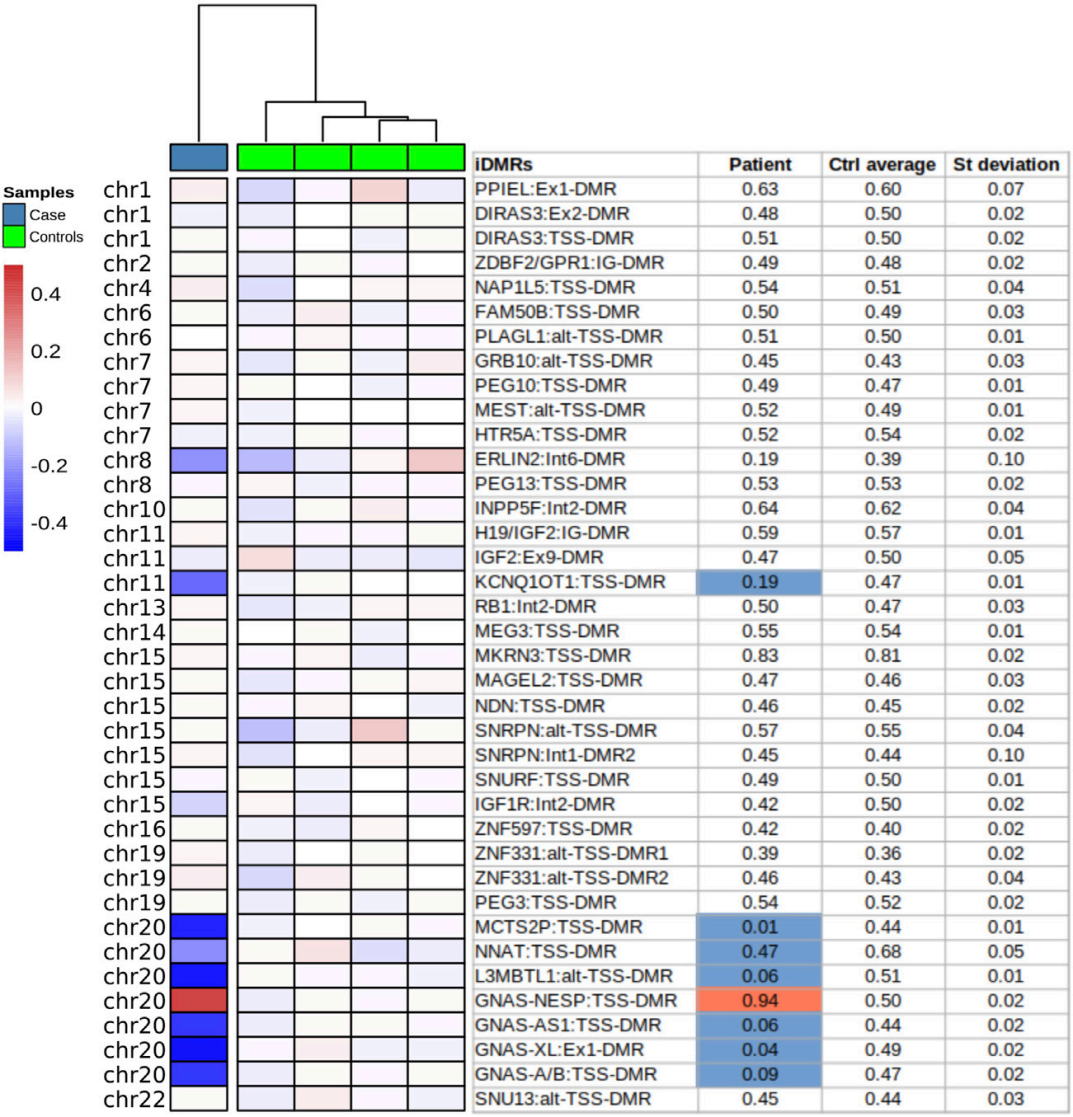
Imprinted DMRs methylation analysis by methylome-array. Methylation of 39 iDMRs analysed by methylome Illumina Epic array on PBL DNA of the proband (Case) and four unaffected individuals (Controls). The methylation profile of each iDMR was calculated as the mean of covered CpGs across the region. Only the regions with at least 4 CpGs were selected. The Beta-values are normalized by the mean of the controls (ΔB-values). In the table, the values exceeding ±3 standard deviation and ±10% of the mean of controls are considered defective and depicted in blue (−) or red (+).

**FIGURE 3:**
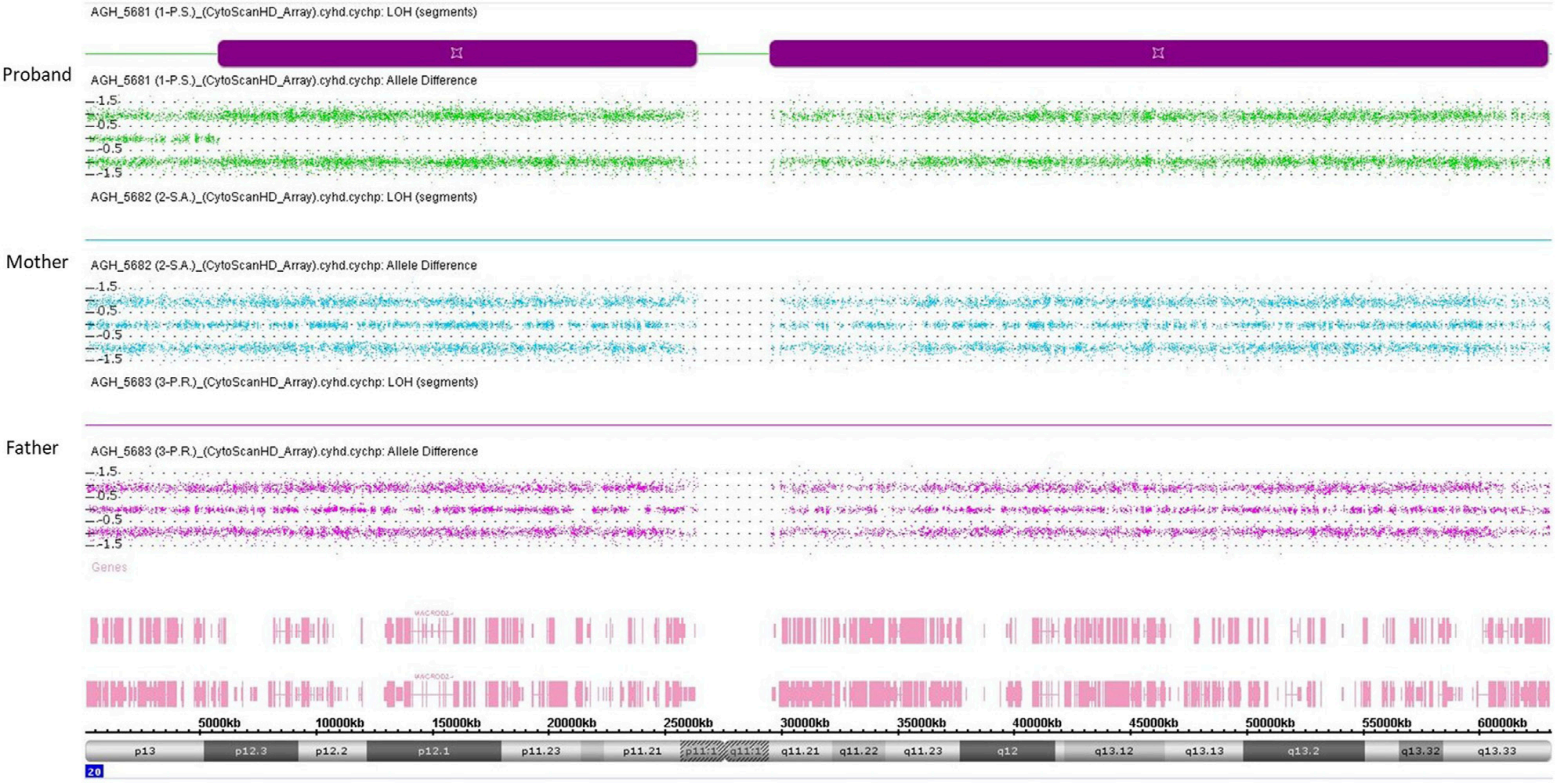
SNP-array analysis on genomic DNA of the proband and his parents. Each of the three panels represents the SNP-array results of chromosome 20 only, indicating the B allele frequency for each SNP. Each point represents a SNP interrogated by “A” + “B” allele probes. Analysis of the allelic differentiation reveals the genotype: “AA” = 1, “AB” = 0, “BB” = −1.

The first molecular diagnosis was obtained in infancy by MS-MLPA using the Probemix ME030 BWS/SRS, that revealed LoM of IC2. A second molecular diagnosis was obtained in childhood by MS-MLPA using the Probemix ME034-B1 Multi-locus. Copy number was normal, but methylation analysis revealed severe LoM of *GNAS-A/B*:TSS-DMR, *GNAS-XL*:Ex1-DMR, *GNAS*-*AS1*:TSS-DMR, and severe GoM of *GNAS-NESP*:TSS-DMR, in addition to the IC2 LoM, consistent with positive diagnoses of both BWS and PHP1B (Figure 1). To investigate if further imprinted loci were affected, the methylation status of 39 iDMRs was determined by employing the Illumina Infinium EPIC methylation array. The methylome results confirmed the MS-MLPA results and revealed further methylation defects in all the iDMRs of chromosome 20 but no methylation changes in the iDMRs located on the other chromosomes. In particular, complete LoM was detected at the *MCTS2P*:TSS-DMR, *NNAT*:TSS-DMR and *L3MBTL1*:alt-TSS-DMR that are all maternally methylated DMRs (Figure 2). These findings were suggestive of upd(20)pat. To corroborate this hypothesis, a SNP-array analysis was performed on genomic DNA of the proband and his parents. The analysis of the proband DNA showed the presence of a long region of copy-neutral loss of heterozygosity (or isodisomy) extending for the entire chromosome 20 except for the telomeric part of the short arm that showed heterodisomy for about 6 Mb (Figure 3). The parental DNAs revealed that both copies of the proband’s chromosome 20 were of paternal origin (Supplementary Table S2).

In conclusion, the molecular analysis revealed IC2 LoM at 11p15.5 and paternal heterodisomy of chromosome 20 (patUPhD20) demonstrating the presence of molecular defects causative of both BWS and PHP1B in our patient. Moreover, detection of 39 iDMRs did not reveal any methylation change in iDMRs other than those of chromosome 11p15 and chromosome 20. Thus, although two ImpDis-associated loci were involved, the criteria for the definition of MLID were not met as the methylation change of one of two affected loci was secondary to a genetic lesion.

## 4 Discussion

The co-existence of two ImpDis is a rare but not negligible phenomenon that is usually associated with MLID. Three cases of BWS and PHP1B co-occurrence have been described so far, all of them related to MLID. We report and discuss here the fourth case of BWS/PHP1B co-occurrence showing pathogenetic mechanisms of disease different from the previously reported cases.

LoM of the 11p15.5 IC2 and *GNAS* loci with no evidence of UPD were found in two of the three previously reported cases (case 1 and case 2 in Table 1), suggesting that the two ImpDis have arisen as consequence of MLID (Bakker et al., 2015; Sano et al., 2016). In the third case (case 3 in Table 1), the methylation defects at 11p15.5 and 20q13.32 appear to arise independently, as consequences of MLID (together with additional loci such as the ImpDis-associated iDMR PLAGL1) and paternal isodisomy of the whole chromosome 20 (patUPiD20), respectively (Choufani et al., 2021).

**TABLE 1:** Molecular and clinical features of the cases with co-occurrence of BWS and PHP1B.

	Case 1	Case 2	Case 3	Proband
Sex	F	F	M	M
Clinical features of BWS	Macrosomia, large umbilical hernia	Macroglossia, umbilical hernia, right hemihyperplasia, transient neonatal hypoglycemia, postnatal overgrowth	Macroglossia, macrosomia, large umbilical hernia, hypoglycemia, ear crease	Macroglossia, macrosomia umbilical hernia, hypoglycemia, chest asymmetry, heterometry of lower limb
Clinical score of BWS	2	6	6	7
Clinical features of PHP1B	Hypocalcemia, hyperphosphatemia, hyperparathyroidemia, PTH resistance, fatigue, no AHO	Hypocalcemia, hyperphosphatemia, PTH resistance, no AHO	Hypocalcemia, hyperphosphatemia, PTH resistance, reduced growth velocity, mild learning disability	Hypocalcemia, hyperphosphatemia, PTH resistance, slow development of language
Methylation defects at BWS (11p15.5) and PHP1B (20q13.32) loci	BWS: LOM-IC2	BWS: LOM-IC2	BWS: LOM-IC2	BWS: LOM-IC2
	PHP1B: LOM-*AS*, *XL*, *A/B*	PHP1B: LOM-*AS*, *XL*, *A/B*	PHP1B: LOM-*AS*, *XL*, *A/B*	PHP1B: LOM-AS, XL, A/B
	GOM-*NESP*	GOM-*NESP*	GOM-*NESP*	GOM-NESP
	(Methylation analysis of IC2 by methylation-sensitive restriction digestion, GNAS locus by MS-MLPA)	(Methylation analysis of multiple imprinted loci by bisulfite pyrosequencing and methylome array)	(Methylation analysis of multiple imprinted loci by MS-MLPA, bisulfite pyrosequencing and methylome array)	(Methylation analysis of multiple imprinted loci by MS-MLPA and methylome array)
Further DMRs affected by methylation defects MLID	Not examined	LOM-*PEG1/MEST, RB1, DIRAS3, FAM50B*	LOM-*DIRAS3, PLAGL1*	LOM-MCTS2P, NNAT, L3MBTL1
	Not examined	Yes	Yes	NO
PatUPD20	Not examined	No	patUPiD20	patUPhD20 SNP-array
		SNP array	SNP-array	
		Microsatellite analyses	Microsatellite analysis	
References	Bakker et al. (2015)	Sano et al. (2016)	Choufani et al. (2021)	This study

Differently from all the above-mentioned cases, LoM occurs only at the 11p15.5 IC2, and differently from case 3 the paternal UPD of the whole chromosome 20 is a heterodisomy rather than an isodisomy in our patient. Whole-chromosome UPiD and UPhD result from different mechanisms (Eggermann et al., 2015). While patUPiD is mainly the product of zygotic monosomy rescue by endoduplication of the paternal chromosome, patUPhD results from trisomy rescue by loss of the maternal chromosome. Therefore, patUPiD rescues an aneuploidy originated during maternal gametogenesis (nullisomic oocyte) and patUPhD rescues an aneuploidy originated during paternal gametogenesis (disomic sperm). Instead, the mosaic form of IC2 LoM indicates its occurrence as post-zygotic event in all cases. It is intriguing that such rare events may occur independently in the same individual. MLID has been associated with either maternal or zygotic gene variants (Eggermann et al., 2022; Pignata et al., 2022). Because most of the chromosome 20 is completely isodisomic in case 3 and mostly isodisomic in our patient (Figure 3), it is possible that a recessive mutation unmasked by the isodisomy may interfere with maintenance of imprinted methylation in both cases 3 and 4. Thus, although the most probable hypothesis is the independent etiology of UPD20 and IC2 LoM, it is not possible to exclude that these two rare events are interconnected. Although, a direct link between GNAS and IC2 is lacking, a few studies have provided evidence for common regulatory mechanisms. For instance, the two zinc-finger proteins ZFP57 and ZFP445 are able to bind both DMRs and maintain their methylation on the maternal allele, as Zfp57/Zfp445 zygotic inactivation result in their loss of methylation in mouse and in human embryonic stem cells (Quenneville et al., 2011; Takahashi et al., 2019). Also, a meta-analysis of micro-array data reveals that genes of both the IC2 and GNAS loci are members of an imprinted gene network controlling embryonic growth in mice (Varrault et al., 2006).

Despite the different molecular mechanisms underlying the four cases of co-occurrence of BWS and PHP1B described so far, their clinical features appear similar and resulting from the sum of BWS-specific and PHP1B-specific characteristics (Table 1). In particular, macrosomia and umbilical hernia were present in all the 4 cases, macrosomia and hypoglycemia in three of them, lateralized overgrowth in two. Mild intellectual disability affected only the cases with pat(20)upd suggesting that additional loci of chr 20 may be involved in the etiology of this clinical feature. As the previously reported cases, our patient did not experience hypocalcemic tetany or seizures, but differently from them, PHP1B was diagnosed at the age of 5 years and not during adolescence.

In conclusion, this study describes the first case of co-existence of BWSp and PHP1B not associated with MLID. We found 11p15.5 IC2 LoM as molecular cause of BWSp, and paternal UPhD20 as cause of PHP1B. As the former is a postzygotic epigenetic defect and the latter occurs as zygotic trisomy rescue by loss of maternal chromosome 20, the most obvious interpretation is that the two ImpDis have distinct and independent etiologies in our patient. However, further studies will tell if the mechanisms causing the epigenetic defect and the UPD may possibly be interconnected, which may explain why they occurred in the same individual. Moreover, our study adds further evidence to the importance of multilocus molecular testing in ImpDis to reveal not only MLID profiles but also complex combinations of imprinted defects, particularly in the cases with atypical clinical presentations.

## Data Availability

The datasets presented in this study can be found in online repositories. The names of the repository/repositories and accession number(s) can be found in the article/Supplementary Material.

## References

[B1] Bakker B. Sonneveld L. J. Woltering M. C. Bikker H. Kant S. G. (2015). A girl with beckwith-wiedemann syndrome and pseudohypoparathyroidism type 1B due to multiple imprinting defects. J. Clin. Endocrinol. Metab. 100, 3963–3966. 10.1210/jc.2015-2260 26367199

[B2] Bastepe M. Altug-Teber O. Agarwal C. Oberfield S. E. Bonin M. Jüppner H. (2011). Paternal uniparental isodisomy of the entire chromosome 20 as a molecular cause of pseudohypoparathyroidism type Ib (PHP-Ib). Bone 48, 659–662. 10.1016/j.bone.2010.10.168 20965295 PMC3039090

[B3] Bliek J. Verde G. Callaway J. Maas S. M. De Crescenzo A. Sparago A. (2009). Hypomethylation at multiple maternally methylated imprinted regions including PLAGL1 and GNAS loci in Beckwith-Wiedemann syndrome. Eur. J. Hum. Genet. 17, 611–619. 10.1038/ejhg.2008.233 19092779 PMC2986258

[B4] Boonen S. E. Pörksen S. Mackay D. J. Oestergaard E. Olsen B. Brondum-Nielsen K. (2008). Clinical characterisation of the multiple maternal hypomethylation syndrome in siblings. Eur. J. Hum. Genet. 16, 453–461. 10.1038/sj.ejhg.5201993 18197189

[B5] Brioude F. Kalish J. M. Mussa A. Foster A. C. Bliek J. Ferrero G. B. (2018). Expert consensus document: Clinical and molecular diagnosis, screening and management of beckwith-wiedemann syndrome: An international consensus statement. Nat. Rev. Endocrinol. 14, 229–249. 10.1038/nrendo.2017.166 29377879 PMC6022848

[B6] Carli D. Riberi E. Ferrero G. B. Mussa A. (2020). Syndromic disorders caused by disturbed human imprinting. J. Clin. Res. Pediatr. Endocrinol. 12, 1–16. 10.4274/jcrpe.galenos.2019.2018.0249 PMC7127890 30968677

[B7] Choufani S. Ko J. M. Lou Y. Shuman C. Fishman L. Weksberg R. (2021). Paternal uniparental disomy of the entire chromosome 20 in a child with beckwith-wiedemann syndrome. Genes (Basel) 12, 172. 10.3390/genes12020172 33513760 PMC7911624

[B8] Colson C. Decamp M. Gruchy N. Coudray N. Ballandonne C. Bracquemart C. (2019). High frequency of paternal iso or heterodisomy at chromosome 20 associated with sporadic pseudohypoparathyroidism 1B. Bone 123, 145–152. 10.1016/j.bone.2019.03.023 30905746 PMC6637416

[B9] Eggermann T. Prawitt D. (2022). Further understanding of paternal uniparental disomy in Beckwith-Wiedemann syndrome. Expert Rev. Endocrinol. Metab. 17, 513–521. 10.1080/17446651.2022.2144228 36377076

[B10] Eggermann T. Soellner L. Buiting K. Kotzot D. (2015). Mosaicism and uniparental disomy in prenatal diagnosis. Trends Mol. Med. 21, 77–87. 10.1016/j.molmed.2014.11.010 25547535

[B11] Eggermann T. Yapici E. Bliek J. Pereda A. Begemann M. Russo S. (2022). Trans-acting genetic variants causing multilocus imprinting disturbance (MLID): Common mechanisms and consequences. Clin. Epigenetics 14, 41. 10.1186/s13148-022-01259-x 35296332 PMC8928698

[B12] Fernández-Rebollo E. Lecumberri B. Garin I. Arroyo J. Bernal-Chico A. Goñi F. (2010). New mechanisms involved in paternal 20q disomy associated with pseudohypoparathyroidism. Eur. J. Endocrinol. 163, 953–962. 10.1530/EJE-10-0435 20837711

[B13] Garin I. Mantovani G. Aguirre U. Barlier A. Brix B. Elli F. M. (2015). European guidance for the molecular diagnosis of pseudohypoparathyroidism not caused by point genetic variants at GNAS: An EQA study. Eur. J. Hum. Genet. 23, 560. 10.1038/ejhg.2015.40 PMC4666586 25762030

[B14] Grosvenor S. E. Davies J. H. Lever M. Sillibourne J. Mackay D. J. G. Temple I. K. (2022). A patient with multilocus imprinting disturbance involving hypomethylation at 11p15 and 14q32, and phenotypic features of Beckwith-Wiedemann and Temple syndromes. Am. J. Med. Genet. A 188, 1896–1903. 10.1002/ajmg.a.62717 35266280 PMC9310769

[B15] Izzi B. Decallonne B. Devriendt K. Bouillon R. Vanderschueren D. Levtchenko E. (2010). A new approach to imprinting mutation detection in GNAS by Sequenom EpiTYPER system. Clin. Chim. Acta 411, 2033–2039. 10.1016/j.cca.2010.08.034 20807523

[B16] Mackay D. J. Boonen S. E. Clayton-Smith J. Goodship J. Hahnemann J. M. Kant S. G. (2006a). A maternal hypomethylation syndrome presenting as transient neonatal diabetes mellitus. Hum. Genet. 120, 262–269. 10.1007/s00439-006-0205-2 16816970

[B17] Mackay D. J. Hahnemann J. M. Boonen S. E. Poerksen S. Bunyan D. J. White H. E. (2006b). Epimutation of the TNDM locus and the Beckwith-Wiedemann syndrome centromeric locus in individuals with transient neonatal diabetes mellitus. Hum. Genet. 119, 179–184. 10.1007/s00439-005-0127-4 16402210

[B18] Mantovani G. Bastepe M. Monk D. de Sanctis L. Thiele S. Usardi A. (2018). Diagnosis and management of pseudohypoparathyroidism and related disorders: First international consensus statement. Nat. Rev. Endocrinol. 14, 476–500. 10.1038/s41574-018-0042-0 29959430 PMC6541219

[B19] Maupetit-Méhouas S. Azzi S. Steunou V. Sakakini N. Silve C. Reynes C. (2013). Simultaneous hyper- and hypomethylation at imprinted loci in a subset of patients with GNAS epimutations underlies a complex and different mechanism of multilocus methylation defect in pseudohypoparathyroidism type 1b. Hum. Mutat. 34, 1172–1180. 10.1002/humu.22352 23649963

[B20] Monk D. Mackay D. J. G. Eggermann T. Maher E. R. Riccio A. (2019). Genomic imprinting disorders: Lessons on how genome, epigenome and environment interact. Nat. Rev. Genet. 20, 235–248. 10.1038/s41576-018-0092-0 30647469

[B21] Ochoa E. Lee S. Lan-Leung B. Dias R. P. Ong K. K. Radley J. A. (2022). ImprintSeq, a novel tool to interrogate DNA methylation at human imprinted regions and diagnose multilocus imprinting disturbance. Genet. Med. 24, 463–474. 10.1016/j.gim.2021.10.011 34906518

[B22] Pignata L. Cecere F. Verma A. Hay Mele B. Monticelli M. Acurzio B. (2022). Novel genetic variants of KHDC3L and other members of the subcortical maternal complex associated with Beckwith-Wiedemann syndrome or Pseudohypoparathyroidism 1B and multi-locus imprinting disturbances. Clin. Epigenetics 14, 71. 10.1186/s13148-022-01292-w 35643636 PMC9148495

[B23] Quenneville S. Verde G. Corsinotti A. Kapopoulou A. Jakobsson J. Offner S. (2011). In embryonic stem cells, ZFP57/KAP1 recognize a methylated hexanucleotide to affect chromatin and DNA methylation of imprinting control regions. Mol. Cell 44, 361–372. 10.1016/j.molcel.2011.08.032 22055183 PMC3210328

[B24] Sano S. Matsubara K. Nagasaki K. Kikuchi T. Nakabayashi K. Hata K. (2016). Beckwith-wiedemann syndrome and pseudohypoparathyroidism type ib in a patient with multilocus imprinting disturbance: A female-dominant phenomenon? J. Hum. Genet. 61, 765–769. 10.1038/jhg.2016.45 27121328

[B25] Takahashi N. Coluccio A. Thorball C. W. Planet E. Shi H. Offner S. (2019). ZNF445 is a primary regulator of genomic imprinting. Genes Dev. 33, 49–54. 10.1101/gad.320069.118 30602440 PMC6317318

[B26] Varrault A. Gueydan C. Delalbre A. Bellmann A. Houssami S. Aknin C. (2006). Zac1 regulates an imprinted gene network critically involved in the control of embryonic growth. Dev. Cell 11, 711–722. 10.1016/j.devcel.2006.09.003 17084362

