# Supplementary material for: Co-occurrence of Beckwith-Wiedemann syndrome and pseudohypoparathyroidism type 1B: coincidence or common molecular mechanism?
Source: Front Cell Dev Biol. 2023 Aug 10;11:1237629. doi: 10.3389/fcell.2023.1237629 (PMC10448386; doi:10.3389/fcell.2023.1237629)
Supplement: Supplementary file 2 [file Table1.DOC]

**Table S1.** Schematic representation of Ca/P metabolism assessment in the patient: gradual improvement after the start of the therapy is evident.

| Data | Ca  mg/ dl | P  mg/dl | PTH  pg/ml | Vit D  ng/ml | ALP  U/l |
| --- | --- | --- | --- | --- | --- |
| Baseline* | 9.4 | 4.5 | n.d. | n.d. | 144 |
| +1y | 8.1 | 6.0 | n.d. | n.d. | 238 |
| +1.5y** | 7.9 | 6.6 | 619.90 | 22.18 | 221 |
| +2y | 8.4 | 5.5 | 486.7 | 30.65 | 202 |
| +2.5y | 9.0 | 5.1 | 199.20 | 35.00 | 195 |
| +3y | 9.1 | 5.0 | 81.90 | 36.78 | 135 |
| +3.5y | 9.8 | 4.6 | 33.90 | 36.48 | 128 |

*3 years old

**start therapy

ALP, Alkaline Phosphatase; PTH, Parathormone

Normal Values: Ca 8.8-10.4 mg/dl, P 2.5-4.5 mg/dl, PTH 10-70 pg/ml, Vitamin D 20-40 ng/ml ALP 110-550 U/L
